# Complete mitochondrial genomes of three *Mangifera* species, their genomic structure and gene transfer from chloroplast genomes

**DOI:** 10.1186/s12864-022-08383-1

**Published:** 2022-02-19

**Authors:** Yingfeng Niu, Chengwen Gao, Jin Liu

**Affiliations:** 1 Yunnan Institute of Tropical Crops, Xishuangbanna, 666100 China; 2 The Affiliated Hospital of Qingdao University, Qingdao, 266400 China

**Keywords:** *Mangifera*, Mitochondrial genome, Gene transfer, Phylogenetic analysis

## Abstract

**Background:**

Among the *Mangifera* species, mango (*Mangifera indica*) is an important commercial fruit crop. However, very few studies have been conducted on the *Mangifera* mitochondrial genome. This study reports and compares the newly sequenced mitochondrial genomes of three *Mangifera* species.

**Results:**

*Mangifera* mitochondrial genomes showed partial similarities in the overall size, genomic structure, and gene content. Specifically, the genomes are circular and contain about 63–69 predicted functional genes, including five ribosomal RNA (rRNA) genes and 24–27 transfer RNA (tRNA) genes. The GC contents of the *Mangifera* mitochondrial genomes are similar, ranging from 44.42–44.66%. Leucine (Leu) and serine (Ser) are the most frequently used, while tryptophan (Trp) and cysteine (Cys) are the least used amino acids among the protein-coding genes in *Mangifera* mitochondrial genomes. We also identified 7–10 large chloroplast genomic fragments in the mitochondrial genome, ranging from 1407 to 6142 bp. Additionally, four intact mitochondrial tRNAs genes (*tRNA-Cys, tRNA-Trp, tRNA-Pro,* and *tRNA-Met*) and intergenic spacer regions were identified. Phylogenetic analysis based on the common protein-coding genes of most branches provided a high support value.

**Conclusions:**

We sequenced and compared the mitochondrial genomes of three *Mangifera* species. The results showed that the gene content and the codon usage pattern of *Mangifera* mitochondrial genomes is similar across various species. Gene transfer from the chloroplast genome to the mitochondrial genome were identified. This study provides valuable information for evolutionary and molecular studies of *Mangifera* and a basis for further studies on genomic breeding of mango.

**Supplementary Information:**

The online version contains supplementary material available at 10.1186/s12864-022-08383-1.

## Introduction

Mitochondria are semi-autonomous organelles found in virtually all eukaryotic cells, and their main function is to generate adenosine triphosphate (ATP) [1]. With the rapid development of genome assembly and sequencing techniques, various complete organellar genomes are being assembled. According to GenBank Organelle Genome Resources (https://www.ncbi.nlm.nih.gov/genome/browse/), the complete mitochondrial genomes currently assembled are less than one-tenth of the chloroplast genomes, indicating that mitochondrial genome assembly may be difficult and complex. Plant mitochondrial genomes are large and variable in size [2, 3], and their structural heterogeneity and gene sequences have meager base substitution rates [4, 5]. The sequences generally have large repeats that mediate recombinational isomerization within a species [4] and numerous non-tandem repeats of 50 bp and up that mediate recombination sometimes within a species, and likely the rearrangements seen between species [6, 7]. The evolution of the mitochondrial genome involves many structural rearrangements and gene transfer [8, 9]. An important feature of plant mitochondrial genome evolution is the transfer of genes between the mitochondria and the chloroplast genomes [10, 11]. Therefore, tracking the transfer of genes between organellar genomes is essential for understanding the evolution of plant mitochondrial genomes.

Among the genus *Mangifera*, mango (*M. indica*) is an important tropical fruit [12, 13], native to tropical and subtropical regions of Southeast Asia [14, 15]. Mango has a wide cultivation range [16], high nutritional value, esthetic appearance, and unique flavor [17]. Thus, it is well accepted by consumers, earning a reputation of “king of tropical fruits” [18]. However, very few studies have been conducted on the *Mangifera* mitochondrial genome. To date, the complete mitochondrial genome of only one *Mangifera* species has been deposited in GenBank [19].

In this study, the mitochondrial genomes of three *Mangifera* species were sequenced and compared with *M. indica* [19]. This study aimed to: (1) comparatively analyze the mitochondrial genome structures of four *Mangifera* species*;* (2) assess gene transfers between the chloroplast and mitochondrial genomes; (3) explore the evolutionary relationships among the *Mangifera* species based on the protein-coding genes of the mitochondrial genome.

## Materials and methods

### Plant material and sequencing

Fresh leaves of the three *Mangifera* species (*M. persiciformis*, *M. longipes*, and *M. sylvatica*) were collected from Xishuangbanna Tropical Flowers and Plants Garden. Total genomic DNA was extracted from all samples using cetyltrimethylammonium bromide (CTAB) method [20]. DNA samples were stored at − 80 °C until use.

About 5–10 μg of total DNA from each sample was used to construct a shotgun library with an average insertion size of 350 bp. Illumina Novaseq6000 (Illumina, USA) was used to sequence the DNA samples in the paired-end sequencing mode. The generated mitochondrial genome raw reads were approximately 0.7–1.2 Gb per sample. Meanwhile, the mitochondrial genome depth of coverage was more than 900 × .

### Mitochondrial genome assembly, annotation, and sequence analysis

The raw sequencing data were filtered using Trimmomatic v0.38 [21], and the SPAdes v3.5.0 was used to de novo assembled with different K-mer parameters [22]. For the regions with complex structures or low sequencing coverage, polymerase chain reaction (PCR) with Sanger sequencing was used for validation to ensure that the circular genome sequence was complete and accurate.

Mitochondrial genome annotation was performed using BLASTN and BLASTX alignment tools of the National Center for Biotechnology Information (NCBI) database (https://www.ncbi.nlm.nih.gov/) using Dicotyledoneae species mitochondrial genomes as the reference sequences. The tRNAs were predicted using the tRNAscan-SE 2.0 [23] software, and their secondary structure maps were generated using ARWEN [24]. A circular diagram of the mitochondrial genomes of *Mangifera* was drawn using OGDRAW v1.3.1 [25].

MEGA v7.0.26 was used to calculate the amino acid composition of the protein-coding genes and their relative synonymous codon usage (RSCU) values [26]. The GC and AT skews were calculated according to the formula: GC skew = (G − C)/(G + C), AT skew = (A − T)/(A + T). Meanwhile, simple sequence repeats (SSR) were detected using MISA software (http://pgrc.ipk-gatersleben.de/misa/misa.html) [27]. Analysis of non-tandem repeats in mitochondrial genomes larger than 50 bp was performed using REPuter [28].

### Identification of chloroplast gene insertion in the mitochondrial genome

We mapped the mitochondrial genome to the plastid genome using the BLASTN tool with the default settings. Circos v0.69 [29] software was then used to map the mitochondrial and chloroplast genomes and gene transfer segments of *Mangifera* plants.

### Phylogenetic analysis

Phylogenetic analyses were performed for the 15 Dicotyledoneae species using *Carica papaya* as outgroups. Protein-coding genes common to the mitochondrial genomes of all species were extracted to construct phylogenetic trees. MUSCLE v.3.8.31 [30] was used to align the mitochondrial gene sequences of the Dicotyledoneae species. Phylogenetic analysis was performed using the maximum likelihood (ML) method via RAxML v8.1.5 with 1000 bootstrap replicates [31].

## Results

### Basic characteristics of the *Mangifera* mitochondrial genomes

Raw sequence data were obtained from *M. longipes* (MZ751075), *M. persiciformis* (MZ751076), and *M. sylvatica* (MZ751077)*.* The three newly sequenced *Mangifera* mitochondrial genomes have been deposited in the GenBank database.

**Fig. 1:**
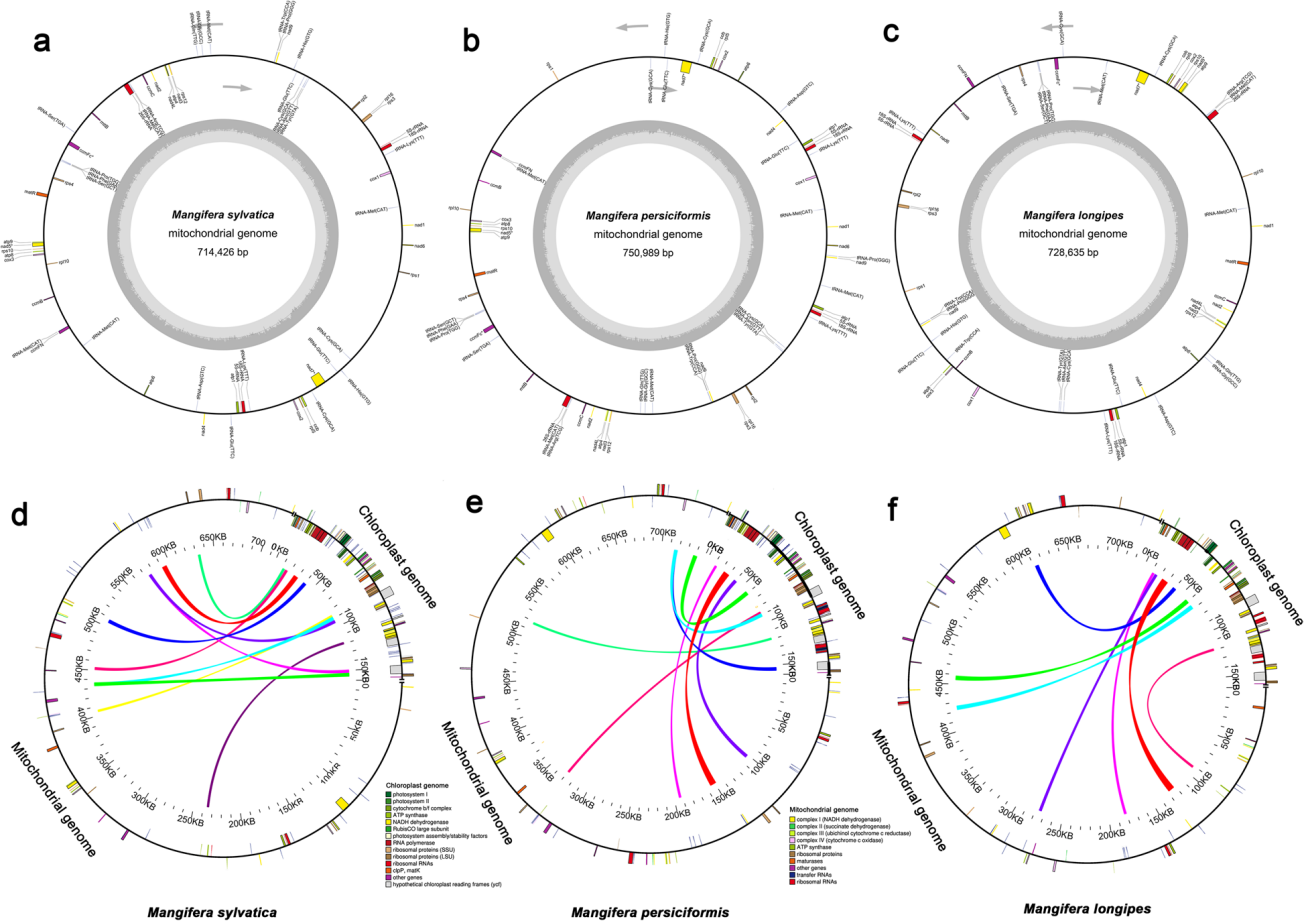
Sequence diagram of *Mangifera* mitochondrial genomes (**a**-**c**), and schematic representation of gene transfers between chloroplast and mitochondrial genomes in *Mangifera* species (**d**-**f**). The chloroplast and mitochondrial genomes used for alignment were from the same species [32]. Genes within a circle are transcribed clockwise, while those outside the circle are transcribed counterclockwise. Colored lines within the circle show the points of chloroplast genome insertion in the mitochondrial genome. (The assembly may best fit on a circular map, but there is no evidence for a circular genomic molecule so far)

The mitochondrial genome sizes of *M. longipes*, *M. persiciformis*, and *M. sylvatica* were 728,635 bp, 750,898 bp, and 714,426 bp, respectively (Fig. 1a-c; Table 1). Furthermore, the GC contents of the various *Mangifera* mitochondrial genomes were similar, ranging from 44.42–44.66%. The four *Mangifera* mitochondrial genomes contained 63–69 predicted functional genes, including five ribosomal RNA (rRNA) genes and 24–27 transfer RNA (tRNA) genes (Table 1). The number and type of genes were partially similar among the four *Mangifera* mitochondrial genomes. The three newly sequenced mitochondrial genomes of *Mangifera* species were submitted to GenBank with the accession numbers; MZ751075, MZ751076, and MZ751077.

**Table 1:** Genomic features of *Mangifera* mitochondrial genome

Species Name	Genome Size	Total Gene Content	rRNA	tRNA	GC (%)	Accession no.
*M. indica*	871,458	69	5	26	44.42	CM021857
*M. longipes*	728,635	63	5	24	44.60	MZ751075
*M. persiciformis*	750,898	67	5	26	44.62	MZ751076
*M. sylvatica*	714,426	66	5	27	44.66	MZ751077

### Gene transfer between the mitochondrial and chloroplast genomes

*Mangifera* mitochondrial genomes are approximately 4.5–5.5 times larger than the chloroplast genomes. We identified 7–10 large chloroplast genomic fragments in the mitochondrial genomes, including genes and intergenomic regions (Fig. 1d-f; Table S1). These fragments ranged from 1407 to 6142 bp, and the sequences had more than 96% similarity in the mitochondrial and chloroplast genomes. The chloroplast genome segments transferred into the mitochondrial genome observed in the three *Mangifera* species included five intact chloroplast genes (*petN, psaA, atpI, trnI-CAU,* and *trnC-GCA*) and many partial genes and intergenic spacers. Four mitochondrial tRNAs genes (*tRNA-Cys, tRNA-Trp, tRNA-Pro,* and *tRNA-Met*) and intergenic spacer regions were also identified (Table S1).

### Codon usage and AT-skew analysis of the protein-coding genes

**Fig. 2:**
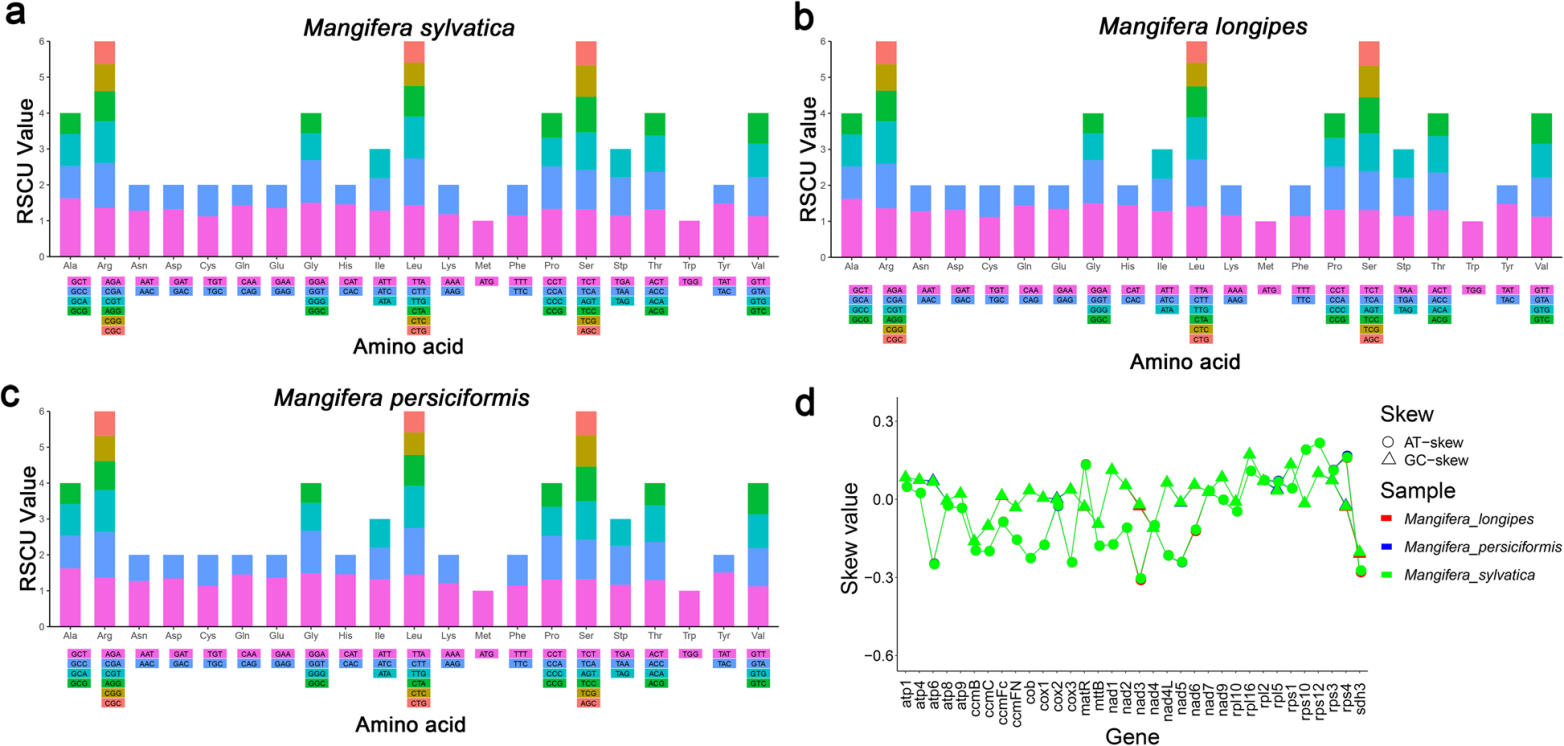
Relative synonymous codon usage (RSCU) (**a**-**c**) and AT-skew analysis of the protein-coding genes (**d**) in the three *Mangifera* mitochondrial genomes

Relative synonymous codon usage (RSCU) analysis of the *Mangifera* mitochondrial genomes are shown in Fig. 2a-c, indicating that all codons are present in the protein-coding genes. The 34–36 protein-coding genes of the three newly sequenced *Mangifera* mitochondrial genomes contain 9401–10,102 codons (Table S2, S3, S4). The results showed that A or T nucleotides were used in high frequency in the third codon position compared to other nucleotides. The most frequent codons used were TTT (Phe), ATT (Ile), and GAA (Glu) and were used ≥278 times in the protein-coding genes of the three newly sequenced *Mangifera* mitochondrial genomes. In contrast, codons with a third codon G or C were used rarely (≤ 73), such as TGC (Cys), CAC (Arg) and TAC (Tyr). This may be a kind of AT mutation bias that has an obvious influence on codon. Notably, the codon usage pattern was highly consistent across the *Mangifera* mitochondrial genomes. The protein-coding genes of the majority strands showed positive AT and negative GC skews, while those of the minority strands showed positive GC and negative AT skews. The AT skews of the protein-coding genes was highly consistent across the *Mangifera* mitochondrial genomes (Fig. 2d; Table S5).

### Repeat sequence analysis of the *Mangifera* mitochondrial genomes

A total of 82–85 SSRs were identified in the *Mangifera* mitochondrial genomes. Mononucleotide repeats of A/T were more prevalent than the other repeat types, dinucleotide repeats (AT/TA) were the second most numerous, while tri- and hexa-nucleotide repeats were less numerous and occurred only in intergenic or intronic regions (Table S6, S7, S8). Besides SSRs, 56–76 non-tandem repeats with lengths ≥50 bp (total length: 26,115–32,557 bp; 3.5–4.5% of the genome) were also identified in the *Mangifera* mitochondrial genomes (Table S9, S10, S11).

### Phylogenetic relationship among the *Mangifera* species

**Fig. 3:**
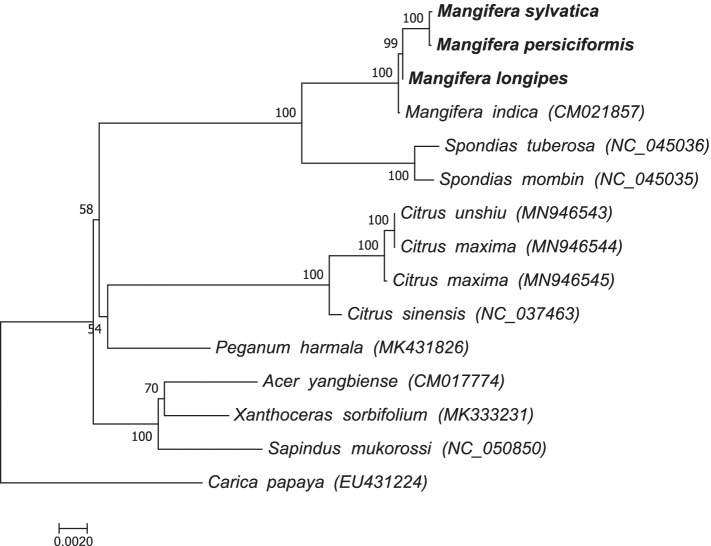
ML phylogenetic tree of four *Mangifera* species with 11 species in Dicotyledoneae based on the common protein-coding genes. Numbers related to the branches are ML bootstrap values

The protein-coding genes common to the mitochondrial genomes of the Dicotyledoneae species were used to infer the phylogenetic location of *Mangifera* species. The ML trees were constructed based on the 19 shared protein-coding genes (PCGs) (*RPS3, COX1, COX2, COX3, NAD9, CCMFN, CCMFC, CCMC, CCMB, ATP9, ATP1, ATP6, ATP4, RPL16, MTTB, NAD2, NAD4L, NAD6, and NAD7).* **The** ML analyses revealed that most branches had very high support. Within the *Mangifera* genus, *M. sylvatica* was evolutionarily closer to *M. persiciformis* (Fig. 3).

## Discussion

Plant mitochondrial genomes have undergone rapid and tremendous structural changes since the initial endosymbiotic event [33, 34]. Because of this evolutionary pattern, the genomic composition of mitochondria has become complex, making plant mitochondrial genome studies comparatively challenging [35, 36]. Here, we sequenced and assembled mitochondrial genomes of three *Mangifera* species. Because of the high recombination frequency, plant mitochondrial genomes have a dynamic structure with multiple configurations such as major loops, sub loops and linear molecules in mitochondria [7]. In the present study, after the gap-filling step, the three *Mangifera* mitochondrial genomes was assembled into a single, circular molecule. A comparative analysis with the *M. indica* mitochondrial genome revealed that the GC content of the four mitochondrial genomes is similar among the *Mangifera* species [19]. Moreover, the rRNA (represented by three genes; 5S subunits, small 18S, and large 26S) and tRNA varies in number (from 24 to 27) and origin (mitochondrial or chloroplast) in the *Mangifera* mitochondrial genomes. The mitochondrial genomes of the common ancestor of angiosperms consist of 41 protein-coding genes [37]. About 34–38 genes of the 41 protein-coding genes were detected in the *Mangifera* mitochondrial genomes, implying that the deleted relevant genes might have been transferred to the nuclear genome, a common phenomenon during angiosperm evolution [38]. Thus, the three newly sequenced *Mangifera* mitochondrial genomes provide new insights into the function and structure of the mitochondrial genomes in *Mangifera* species.

Gene transfer in cells occurs between different organelles, including the chloroplast, mitochondria, and nucleus [39, 40]. Gene transfer from chloroplasts to mitochondrial genomes is common during long-term plant evolution [39, 41], both intracellular gene transfer and horizontal gene transfer are involved in the process of gene transfer between the chloroplast and mitochondrial genomes [42]. Compared to our previous study [32], the three newly mitochondrial genomes provide new findings in respect to gene transfer from the *Mangifera* chloroplast genome to the mitogenome. First, we found that the chloroplast genome segments transferred into the mitochondrial genome observed in the *Mangifera* species were relatively conserved (Fig. 1d-f; Table S1), these chloroplast genome segments transferred to the mitogenome were also found in species of other genera [39, 43]. In addition, we also found that *Mangifera* has a broadly divergent segment location resulting from chloroplast gene insertion into the mitochondrial genome. Intracellular gene transfers may account for the high degree of rearrangements among the mitochondrial genomes [44]. Because the chloroplast genome segments transferred into the mitochondria highly aligned with the original chloroplast genome sequences, such gene transfers might have caused disassembly of the mitochondrial genomes.

The codon usage bias was measured by calculating the relative synonymous codon usage (RSCU). The results indicate a strong A or T bias in the third position of the codon in the protein-coding genes of the *Mangifera* mitochondrial genomes, which is commonly observed in plant mitochondrial genomes [1, 44]. Codon usage pattern in the *Mangifera* mitochondrial genomes were highly consistent.

Plant mitochondrial genomes are rich in repeat sequences [45], the vast majority of differences in the size of plant mitochondrial genomes can be explained by differences in the size of the repeat sequences. SSRs and non-tandem repeats from three newly sequenced *Mangifera* mitochondrial genomes were investigated in this study. SSRs are important molecular markers for species identification, evolutionary analysis and studying genetic diversity [46]. Almost all angiosperm mitochondrial genomes have large (> 1 kb) non-tandem repeats and they are recombinationally active [6]. The longest non-tandem repeat in the mitochondrial genomes of *Mangifera* are 8348–13,655 bp and may be one of the reasons for isomerization. Previous studies have uncovered the genetic relationships among the *Mangifera* species through morphological, nuclear, internal ribosomal transcribed spacer (ITS), amplified fragment length polymorphism (AFLP), and chloroplast gene analyses [32, 47–50]. Plant mitochondria have evolved rapidly, resulting in heterogeneity, large-scale genomic reorganization, and gene mosaicism in the mitochondrial genomes of various species [34, 51]. Size and structural variations of plant mitochondrial genomes are evident, but functional genes remain conserved [36, 52]. Here, sequence-based phylogenetic tree was constructed using the protein-coding genes to explore the evolutionary relationship between *Mangifera* and Dicotyledoneae species. There were several inconsistencies between the chloroplast and mitochondrial phylogenetic trees regarding the phylogenetic topology [32]. Differential inheritance of organelles in the same cytoplasm can disrupt the linkage disequilibrium between mitochondrion and chloroplast [53, 54], and if this occurs, phylogenetic reconstruction of the two organelle genomes could conflict. In addition, limited sampling, incomplete lineage classification, and differences in the evolutionary rates could account for the conflicting phylogenetic reconstruction of the two organelle genomes [55]. Therefore, larger-scale sampling is required better to understand the evolution of the mitochondrial genome of *Mangifera*. Sequencing and assembling the three new complete mitochondrial genomes of *Mangifera* is the first step towards understanding the mitochondrial genome variation in this genus.

## Conclusions

Here, we sequenced and compared the mitochondrial genomes of three *Mangifera* species. The results showed that the gene content and the codon usage pattern was highly consistent across the *Mangifera* mitochondrial genomes. We also identified 7–10 large fragments transferred from the chloroplast genome to the mitochondrial genome. The findings of this study provide valuable genetic resources for further studies on *Mangifera* species.

## Supplementary Information

**Additional file 1.**

## Data Availability

The data supporting the findings of this study are freely available in GenBank on the NCBI website at https://www.ncbi.nlm.nih.gov, using the accession number MZ751075, MZ751076, and MZ751077. Raw sequencing data have been deposited at the NCBI Sequence Read Archive (SRA) under accession PRJNA778602.
